# 2634. All-Cause Hospitalization Following Outpatient Episodes for RSV Among Adults in the United States using an Integrated EHR-Claims Data Source

**DOI:** 10.1093/ofid/ofad500.2246

**Published:** 2023-11-27

**Authors:** Suzanne Landi, Diana Garofalo, Chern Chuan Soo, Amie Scott, Lili Jiang, Jill McCarthy, Margaret Tawadrous, Glenn Pixton, Niki Alami, Scott P Kelly, Joshua T Swan

**Affiliations:** Pfizer, New York, New York; Pfizer, New York, New York; Pfizer, New York, New York; Pfizer, Inc, New York, New York; Pfizer, New York, New York; Quanticate, Montreal, Quebec, Canada; Pfizer, Inc, New York, New York; Pfizer, New York, New York; Pfizer, New York, New York; Pfizer, New York, New York; Pfizer, New York, New York

## Abstract

**Background:**

Respiratory syncytial virus (RSV) can cause severe disease in older adults (≥65 years) or those with comorbidities. Understanding the risk of hospitalization after RSV diagnosis through current real-world data analyses is limited by estimates combining cases identified in inpatient and outpatient settings. To address this gap and to better understand opportunities for intervention to prevent such hospitalizations, we quantified the crude 28-day risk of all-cause hospitalizations following RSV infections initially managed in an outpatient setting.

**Methods:**

This retrospective observational study identified outpatient RSV episodes (clinics and emergency departments [ED]) for RSV between 01Sep2016-31Oct2022 using Optum’s de-identified Integrated Claims-Clinical data set, which contains linked person-level claims and electronic health record (EHR) data. RSV diagnosis was defined using positive test results (PCR, antigen, or culture) and ICD-10-CM diagnosis codes. Comorbidities were identified using diagnosis, procedure, and medication codes. Only the first eligible episode (index date) for each study-defined RSV season (01Oct–30Sep) was included (Figure 1). Episodes hospitalized on the index date or with an RSV-related hospitalization 14 days prior to the index date were excluded. Crude risk of all-cause hospitalization was assessed over a 28-day follow-up period.

Study schematic for the retrospective observational cohort study design

**Figure:**
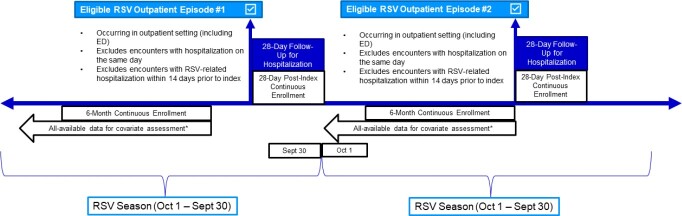

The schematic depicts how eligible RSV outpatient episodes were identified and the inclusion/exclusion criteria that were applied to generate the final sample.

**Results:**

Of 2,792 included episodes, 62% occurred among females, 55% occurred among patients aged ≥65, and 21% received ED care on the index date. Between 2016-2018, the 28-day risk of hospitalization ranged from 6-9% and declined to 3% in 2019/20 (Figure 2). The risk of hospitalization also varied by age or the presence of comorbidities (Table 1).

**Figure:**
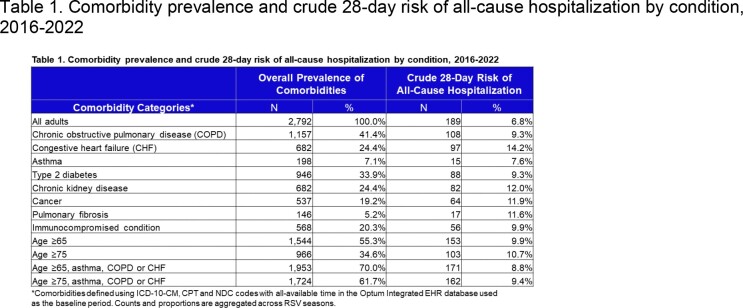

This table summarizes the crude 28-day risk of all-cause hospitalization for adults overall and stratified by clinical conditions and age groups.

**Figure:**
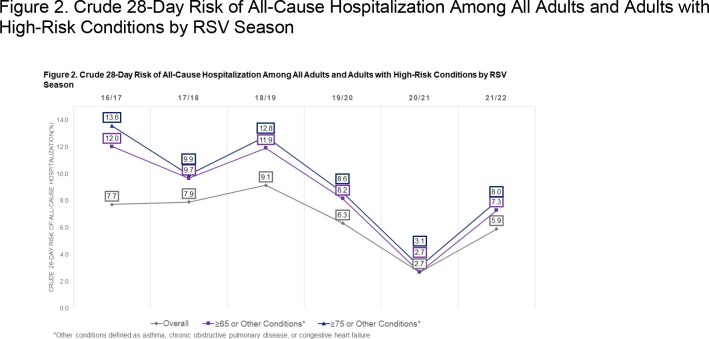

This figure displays the crude 28-day risk of all-cause hospitalization following an outpatient episode of RSV, stratified by season and displayed for adults with high-risk conditions.

**Conclusion:**

A meaningful risk of all-cause hospitalization following outpatient RSV episodes was observed using a US integrated database that captures a patient’s longitudinal healthcare journey. Risk of hospitalization varied across seasons and was influenced by age and comorbidities. Results include diagnosed cases, and RSV infections may have been underreported due to infrequent standard-of-care testing.

**Disclosures:**

**Suzanne Landi, PhD**, Pfizer, Inc: Employment|Pfizer, Inc: Stocks/Bonds **Diana Garofalo, PhD MPH**, Pfizer: Stocks/Bonds **Amie Scott, MPH**, Pfizer, Inc: Employee|Pfizer, Inc: Stocks/Bonds **Jill McCarthy, PhD**, Pfizer: Contractor **Margaret Tawadrous, MD, MS**, Pfizer: Full time employee|Pfizer: Full-time employee|Pfizer: Full-time employee|Pfizer: Full -time employee|Pfizer: Ownership Interest|Pfizer: Ownership Interest|Pfizer: Ownership Interest|Pfizer: Ownership Interest|Pfizer: Stocks/Bonds|Pfizer: Stocks/Bonds|Pfizer: Stocks/Bonds|Pfizer: Stocks/Bonds **Glenn Pixton, MS**, Abbott: Stocks/Bonds|Abbvie: Stocks/Bonds|Pfizer: employee|Pfizer: Stocks/Bonds|Viatris: Stocks/Bonds **Niki Alami, MD**, Pfizer: Employee|Pfizer: Stocks/Bonds **Scott P. Kelly, PhD**, Pfizer: Employee|Pfizer: Stocks/Bonds **Joshua T. Swan, PharmD, MPH, BCPS, FCCM**, CareDx: Grant/Research Support|Genentech: Grant/Research Support|Grifols Share Services North America: Grant/Research Support|Heron Therapeutics: Grant/Research Support|Kedrion Biopharma: Advisor/Consultant|Kedrion Biopharma: Grant/Research Support|Pacira Pharmaceuticals: Grant/Research Support|Pfizer: Grant/Research Support|Pfizer: Employee|Pfizer: Stocks/Bonds|VigiLanz Corporation: Grant/Research Support

